# Major Neutrophil-Derived Soluble Mediators Associate With Baseline Lung Pathology and Post-Treatment Recovery in Tuberculosis Patients

**DOI:** 10.3389/fimmu.2021.740933

**Published:** 2021-11-23

**Authors:** Caleb Nwongbouwoh Muefong, Olumuyiwa Owolabi, Simon Donkor, Salome Charalambous, Joseph Mendy, Isatou C. M. Sey, Abhishek Bakuli, Andrea Rachow, Christof Geldmacher, Jayne S. Sutherland

**Affiliations:** ^1^ Vaccines and Immunity Theme, Medical Research Council (MRC) Unit The Gambia at London School of Hygiene and Tropical Medicine (LSHTM), Fajara, Gambia; ^2^ Division of Infectious Diseases and Tropical Medicine, University Hospital, Ludwig Maximilian University (LMU) Munich, Munich, Germany; ^3^ School of Public Health, Aurum Institute, Johannesburg, South Africa; ^4^ International Clinical Trials Unit, German Centre for Infection Research (DZIF), Partner Site Munich, Munich, Germany

**Keywords:** tuberculosis, neutrophils, myeloperoxidase, S100A8/9, MMP8, lung pathology

## Abstract

**Background:**

The inflammatory response to *Mycobacterium tuberculosis* results in variable degrees of lung pathology during active TB (ATB) with central involvement of neutrophils. Little is known about neutrophil-derived mediators and their role in disease severity at baseline and recovery upon TB treatment initiation.

**Methods:**

107 adults with confirmed pulmonary TB were categorised based on lung pathology at baseline and following successful therapy using chest X-ray scores (Ralph scores) and GeneXpert bacterial load (Ct values). Plasma, sputum, and antigen-stimulated levels of MMP1, MMP3, MMP8, MMP9, MPO, S100A8/9, IL8, IL10, IL12/23(p40), GM-CSF, IFNγ, and TNF were analysed using multiplex cytokine arrays.

**Results:**

At baseline, neutrophil counts correlated with plasma levels of MMP8 (rho = 0.45, p = 2.80E−06), S100A8 (rho = 0.52, p = 3.00E−08) and GM-CSF (rho = 0.43, p = 7.90E−06). Levels of MMP8 (p = 3.00E−03), MMP1 (p = 1.40E−02), S100A8 (p = 1.80E−02) and IL12/23(p40) (p = 1.00E−02) were associated with severe lung damage, while sputum MPO levels were directly linked to lung damage (p = 1.80E−03), Mtb load (p = 2.10E−02) and lung recovery (p = 2.40E−02). Six months of TB therapy significantly decreased levels of major neutrophil-derived pro-inflammatory mediators: MMP1 (p = 4.90E−12 and p = 2.20E−07), MMP8 (p = 3.40E−14 and p = 1.30E−05) and MMP9 (p = 1.60E−04 and p = 1.50E−03) in plasma and sputum, respectively. Interestingly, following H37Rv whole cell lysate stimulation, S100A8 (p = 2.80E−02), MMP9 (p = 3.60E−02) and MPO (p = 9.10E−03) levels at month 6 were significantly higher compared to baseline. Sputum MMP1 (p = 1.50E−03), MMP3 (p = 7.58E−04), MMP9 (p = 2.60E−02) and TNF (p = 3.80E−02) levels were lower at month 6 compared to baseline in patients with good lung recovery.

**Conclusion:**

In this study, patients with severe lung pathology at baseline and persistent lung damage after treatment were associated with higher plasma and sputum levels of major pro-inflammatory neutrophil-derived mediators. Interestingly, low sputum MPO levels were associated with severe lung damage, higher Mtb burden and low recovery. Our data suggest that therapeutic agents which target these mediators should be considered for future studies on biomarkers and host-directed therapeutic approaches against TB-related lung pathology and/or lung recovery.

## Introduction

Tuberculosis (TB) caused 1.2 million deaths from HIV-negative individuals in 2019 (1). While treatment is available, former patients are more likely to experience long-term pulmonary disability (2) and only about 50% of patients achieve full recovery from lung damage (3). It has been suggested that higher initial inflammatory responses against *Mycobacterium tuberculosis* (Mtb) lead to more severe lung damage prior to treatment initiation (4) and thus reduced of recovery following treatment.

Inflammatory mediators generated during the natural immune response to Mtb (5) are linked to increased disease severity, bacterial burden and delayed culture conversion. However, the overall inflammatory response depends on the interplay between pro- and anti-inflammatory mediators (6). Interestingly, reports show that severe inflammation and lung damage following *Mycoplasma pneumoniae* infection in mice may be a result of oxidant–antioxidant imbalances which can be reduced by immunosuppression (6). Similarly, certain neutrophil sub-types have been shown to express immunosuppressive functions including: CD16^bright^CD62L^low^ neutrophils (7) and granulocytic myeloid-derived suppressor cells (G-MDSCs) (8, 9). These support the idea that an equilibrium between neutrophilic pro- and anti-inflammatory functions (10–14) determines the extend of inflammation and lung damage in TB patients.

Previous studies have investigated a broad range of biomarkers for TB disease progression and lung damage severity in humans (15–19), recently reviewed (20–22). While some neutrophilic activities have been tested, major neutrophil-derived mediators have not been the main focus. Neutrophils are mainly pro-inflammatory but recent studies reveal that different subtypes also display anti-inflammatory functions (11, 14, 23, 24) depending on the type and quantity of inflammatory mediators produced. A current challenge is to elucidate which neutrophil subtypes and mediators are predominantly pro- or anti-inflammatory during active TB and to determine the underlying immunological mechanisms involved in protective outcomes. Muefong and Sutherland reviewed (12) promising neutrophil-derived targets for developing host directed therapies (HDTs) against TB-induced lung pathology. We also recently showed, in a smaller group of participants from this Gambian cohort, that immature (banded) neutrophils and IL10-expressing CD16^dim^CD62L^low^ neutrophils are associated with reduced lung damage in active TB patients pre-treatment (13). Additionally, MDSCs are currently considered in the development of HDTs against TB progression and Mtb control (9, 25, 26) due to their role as effectors of Mtb pathogenesis and their modulatory role on T-cell function.

Studies in mice (27), macaques (28, 29) and humans (30, 31) suggest that heightened neutrophil function correlates with tissue injury. For example, during hypoxic conditions, human neutrophils have been shown to drive tissue destruction during ATB by secreting matrix metalloproteinases (MMPs) like MMP8 and MMP9 (32). Sputum MMP levels have also been associated with disease severity in ATB patients pre-treatment (33) and excess MMP activity enhances tissue injury in clinical studies and preclinical models of pulmonary pathology (34). S100A8/9 is another neutrophil-derived mediator known to exacerbate the inflammatory response to Mtb infection and it is currently targeted in Mtb control studies (28, 35).

On the other hand, recent studies highlight an immunoregulatory effect of granulocytes (36). In mice exposed to zymosan, deficiency in myeloperoxidase (MPO)—a major constituent of neutrophil granules—results in severe lung inflammation (37), suggesting that MPO could play immunomodulatory functions; an observation which has not been made in TB. Hence, different neutrophil-related mediators could differentially influence ATB-related lung pathology.

We contribute to the field of TB biomarkers by focusing on major neutrophil-derived inflammatory mediator levels in ATB patients and relate this to chest X-ray (CXR) based lung pathology scores and bacterial load before and after TB therapy. We address gaps in our understanding of TB pathogenesis by monitoring the impact of neutrophil-derived mediators on the severity of TB-induced lung pathology to inform future experiments in controlled animal models investigating TB HDTs.

## Methods

### Ethics Approval

Ethical approval was obtained from the Medical Research Council/Gambia government joint ethics committee (SCC1523). All study participants provided written informed consent prior to collection of samples.

### Participants

Adult, TB patients with positive GeneXpert (Cepheid, USA) results were recruited from the TB clinic at the MRC Unit The Gambia at LSHTM between April 2018 and October 2019 as part of a parent study, TB Sequel (3). This study was conducted on a sub-cohort of TB Sequel and patients were selected based on their lung recovery outcome post-treatment. All participants were later confirmed to have a positive mycobacteria growth indicator tube (MGIT) culture result at baseline, were drug sensitive and had not previously received anti-TB therapy (ATT). They were given standard TB therapy consisting of 2 months intensive phase and 4 months continuation phase. Sputum liquid MGIT culture was performed at baseline (BL), 2 months (2M) and 6 months (6M) after ATT initiation. Heparinised blood and sputum samples were collected and processed at BL, 2M and 6M of standard treatment. All patients were culture negative by 6M and HIV positive patients were excluded from analysis.

### Scoring of Chest Radiographs

Chest radiographs were analysed based on the Ralph score (RS) (38). Briefly, posteroanterior chest radiographs were assessed for the percentage of the lung fields affected by known ATB features. When at least one cavity could be identified, 40 points were added to the value of percentage lung affected. The median [interquartile range (IQR)] RS score at baseline (RS_Med_) of all participants in our cohort was determined, 65 [29–80]. Lung damage severity (pre- and post-treatment) groupings were defined as follows: “mild pathology” (RS < RS_Med_) and “severe pathology” (RS ≥ RS_Med_).

### GeneXpert MTB/RIF Results

The GeneXpert^®^ machine (Cepheid, USA) was used to determine cycle threshold (Ct) values at baseline. The lowest Ct value generated among the five rpoB probes of Xpert MTB/RIF (or of the four rpoB probes in the nested-PCR stage for GeneXpert Ultra) was taken as a measure of the Mtb cell number (39). The median [interquartile range (IQR)] Ct value (Ct_Med_) of all participants was computed, 17.4 [17.1–18.4]. Patients were grouped into: “high Mtb load” (Ct < Ct_Med_) and “low Mtb load” (Ct > Ct_Med_) groups.

### Recovery from Severe Lung Pathology After Treatment

For each patient, RS changes (ΔRS) from BL to 6M (ΔRS = RS at BL/RS at 6M) and median change [IQR] in RS of the entire cohort (ΔRS_Med_) were computed, 6.5 [1.6–14]. Patients were grouped into: “Good” (ΔRS ≥ ΔRS_Med_) and “Poor” (ΔRS < ΔRS_Med_) lung recovery groups. Nine participants had a ΔRS equal to the ΔRS_Med_.

### Sputum Sample Supernatant Collection

Aliquots of sputum were digested with an equal volume of Sputolysin (MerckMillipore, USA) for 15 min and centrifuged. The supernatant was harvested and stored at −80°C until use.

### Whole Blood Processing, Storage and Stimulation

Plasma was obtained from blood vials by centrifugation at 2,500 rpm and stored at −80°C prior to use. Approximately 500 µl of whole blood was stimulated with either ESAT-6/CFP-10 peptide pool (EC; at 2.5 µg/ml/peptide), purified protein derivative (PPD at 10 µg/ml; Staten Serum Institute, Denmark), H37Rv whole cell lysate (WCL; at 10 µg/ml; BEI Resources, USA) or phorbol 12-myristate 13-acetate (PMA; positive control; 10 µg/ml) along with co-stimulatory antibodies (anti-CD28, anti-CD49d; Becton Dickinson, USA); or unstimulated/cultured with medium alone (NIL; negative control). Following overnight incubation at 37°C, 5% CO_2_, plates were spun (1,500 rpm, 5 min) and 200–250 μl of supernatant was harvested from each well into 0.5 ml Sarstedt tubes prior to storage at −80°C for multiplex cytokine assays.

### Multiplex Cytokine Arrays

Multiplex cytokine arrays were performed using a customised 13-plex inflammatory marker panel (R&D Systems, USA) according to the manufacturer’s instructions. The 13 analytes measured were GM-CSF, IL8/CXCL8, IL12/23(p40), MMP3, MMP9, S100A8, S100A9, TNF, IFNγ, IL10, MMP1, MMP8, and MPO. The minimum levels of detection for these were: 11.52, 2.96, 383.13, 78.48, 128.31, 74.86, 8.44, 42.35, 3.70, 40.95, 241.11, 113.00, and 26.87 pg/ml, respectively. Briefly, lyophilised standards were reconstituted and serial dilutions performed. Coupled beads were diluted in assay buffer and 50 μl were added to each well of the assay plate. Approximately 50 μl of diluted standards, blanks, samples (plasma, ag-stimulated supernatants or *ex vivo* sputum) and controls were added per well. Plates were then incubated at room temperature (RT), with shaking (350 rpm, 2 h) followed by three washes in wash buffer. Detection antibodies were diluted in detection antibody diluent as recommended and 50 μl added to each well followed by another 1 h incubation period. Following three washes, 100× streptavidin-PE was diluted in wash buffer (one in 100) and 50 μl added to each well. Plates were then incubated for 30 min and washed three times. Approximately 100 μl of assay buffer were then added to each well, plates were shaken for 2 min and read using Bioplex 200 plate reader with Bio-Plex Manager Software (version 6.1; Bio-Rad, Belgium).

### Data Analysis

All statistical analyses were performed using R version 3.5.2 (40). For antigen-specific blood responses, background was subtracted using the unstimulated (NIL) samples. Non-parametric tests were used for all comparisons. Differences between BL, 2M and 6M samples within each group were analysed using a Kruskal–Wallis test with Dunn’s post-test comparison. For comparisons between severity, treatment response and recovery groups, a Wilcoxon rank sum test was performed. The Benjamini–Hochberg test (41) was used to adjust for multiple comparisons throughout. Adjusted p values (q values) of less than 0.05 were considered statistically significant. Linear regression models were used to determine significant differences after adjusting for sex.

## Results

### Patient Demographics

We analysed pre-selected plasma and sputum samples from 107 adult HIV negative, pulmonary TB patients of which 77% were males (**Table 1**). The median [interquartile range (IQR)] CXR score at baseline was 65 [29–80] with 46 patients in the mild (RS <65) and 61 patients in the severe group (RS ≥65). The median [IQR] CXR score for the mild and severe groups at baseline was 25 [16.2–51.5] and 75 [65–90] respectively. For patients with severe damage at baseline, the median [IQR] change in CXR scores (ΔRS) from baseline to 6 months was 6.5 [1.6–14] with 30 patients in the good recovery (ΔRS ≥6.5) and 22 in the poor recovery group (ΔRS <6.5). Within the good and poor recovery groups, the median [IQR] ΔRS was 14 [9.5–18] and 1.5 [1.3–2.6], respectively. Nine of the patients with severe damage at BL could not be classified into either good or poor recovery groups due to missing month 6 CXR scores (NA). At the end of treatment (6M) the median [IQR] CXR for the mild and severe groups was 5 [0–10] and 5 [5–13.5], respectively. For bacterial load calculations, we analysed the Xpert Ct values for all participants. The median [IQR] Ct value was 17.4 [17.1–18.4] with 45 patients in the high bacterial load group (Ct <17.4) and 53 patients in the low bacterial low group (Ct >17.4). The median [IQR] Ct values for the high and low bacterial load groups were 17.0 [16.8–17.1] and 18.4 [17.8–19.6], respectively. CXR-derived Ralph scores and Xpert MTB/RIF cycle threshold weakly correlated (rho = −0.24, p = 1.40E−02) at baseline. No differences in age were observed in the mild *vs*. severe lung damage, low *vs*. high Mtb load and good *vs*. poor recovery groups (**Table 1**). Male sex was associated with higher levels of lung damage (p = 3.90E−03) and Mtb loads (p = 4.50E−03) but not with reduced lung recovery (ns).

**Table 1:** Patient demographics.

	Total	CXR-defined		GeneXpert-defined			Lung Recovery	
		Mild N = 46	Severe N = 61	Low N = 53	High N = 45	NA N = 9	Good N = 30	Poor N = 22
Age	32 [23–40]	29.5 [21–39]	32 [26–41]	29 [22–40]	31 [25–40]	32 [30–34]	32 [24–41]	32 [26–37]
Male n (%)	82 (77)	29 (63)	53 (87)	34 (64)	40 (89)	8 (89)	26 (87)	19 (86)
		p = 3.90E−03			p = 4.50E−03		ns	

ns, not significant; CXR, chest X-ray; age = median [interquartile range].

### Analysis of ATB Severity

The two measures of ATB severity that we used were sputum GeneXpert Ct values and CXR Ralph scores. There was a weak negative correlation between patient Ct values and Ralph scores at baseline (rho = −0.24, p = 1.40E−02) (**Supplementary Figure 1A**) as previously reported (42). We also observed a weak positive correlation between baseline Ralph scores and neutrophil counts (rho = 0.22, p = 2.50E−02) but not between baseline Ct values and baseline percentage neutrophil counts (**Supplementary Figures 1B, C** **)**.

Neutrophil levels are associated with higher risk of lung damage (10) and death in TB patients (30) and we recently showed that neutrophil activation and function vary in ATB patients based on the severity of the lung pathology (43). Hence, we monitored the impact of neutrophil counts and neutrophilic inflammatory mediator levels on lung damage severity or Mtb burden.

### Association Between Neutrophil Count and Analyte Concentrations in Plasma at Baseline

At baseline, plasma concentrations of all inflammatory mediators, excluding MPO and MMP9, correlated with absolute neutrophil counts (**Table 2**). The strongest correlations were observed for MMP8 (rho = 0.45, p = 2.80E−06), S100A8 (rho = 0.52, p = 3.00E−08), S100A9 (rho = 0.33, p = 6.30E−04) and GM-CSF (rho = 0.43, p = 7.90E−06). Analysis within groups showed that plasma MPO was associated with neutrophil counts in patients with high Mtb load only (rho = 0.37, p = 1.50E−02) and MMP9 was associated with neutrophil counts in patients with severe lung damage only (rho = 0.26, p = 4.10E−02) (**Table 3**). S100A8, MMP8, S100A9, IL10, GM-CSF, TNF, and IFNγ correlated with neutrophil count in patients with both severe lung damage and high Mtb load at baseline (**Table 3**).

**Table 2:** Correlation between neutrophil count and analyte concentrations at baseline.

	rho	p-value
MMP1	0.20	ns
MMP3	0.23	2.20E−02
MMP8	0.45	2.80E−06
MMP9	0.16	ns
MPO	0.021	ns
S1000A8	0.52	3.00E−08
S100A9	0.33	6.30E−04
IL8	0.22	3.00E−02
IL10	0.32	1.30E−03
IL12/23(p40)	0.32	1.10E−03
GM-CSF	0.43	7.90E−06
TNF	0.38	1.00E−05
IFNγ	0.36	2.40E−04

ns, not significant; rho, spearman’s rank correlation coefficient.

**Table 3:** Correlation between neutrophil count and analyte concentrations in plasma for patients with different degree of lung damage (CXR) and Mtb load (GeneXpert) at baseline.

Analyte	Lung damage				Mtb load			
	Mild		Severe		Low		High	
	rho	p-value	rho	p-value	rho	p-value	rho	p-value
MMP1	0.16	ns	0.16	ns	0.20	ns	0.18	ns
MMP3	0.13	ns	0.26	ns	0.26	ns	0.11	ns
MMP8	0.46	2.00E−03	0.40	2.10E−03	0.27	ns	0.65	2.80E−06
MMP9	−0.13	ns	0.26	4.10E−02	0.04	ns	0.29	ns
MPO	−0.13	ns	0.10	ns	−0.18	ns	0.37	1.50E−02
S1000A8	0.39	9.70E−03	0.49	8.20E−05	0.41	3.50E−03	0.59	2.60E−05
S100A9	0.39	1.00E−02	0.27	4.00E−02	0.26	ns	0.47	1.40E−03
IL8	0.06	ns	0.26	4.70E−02	0.09	ns	0.30	ns
IL10	0.28	ns	0.38	3.80E−03	0.20	ns	0.42	6.40E−03
IL12/23(p40)	0.18	ns	0.35	6.40E−03	0.13	ns	0.50	7.20E−04
GM-CSF	0.27	ns	0.50	5.40E−05	0.34	1.50E−02	0.51	5.50E−04
TNF	0.18	ns	0.45	4.10E−04	0.38	7.30E−03	0.37	1.50E−02
IFNγ	0.18	ns	0.43	8.20E−04	0.21	ns	0.46	1.90E−03

ns, not significant; rho, spearman’s rank coefficient.

For patients with mild lung damage at baseline, correlations were weaker and only significant for MMP8, S100A8, and S100A9 (**Table 3**). Likewise, within the low Mtb load group, significant correlations were only observed for S100A8, TNF, and GM-CSF (**Table 3**). These values reveal that while most plasma neutrophilic inflammatory marker levels are generally associated with neutrophil counts irrespective of the severity of lung pathology, MPO and MMP9 are only associated with neutrophil counts in patients with a severe form of lung pathology.

### Analysis of Neutrophil Mediators in Relation to Lung Pathology and Sex at Baseline

**Figure 1:**
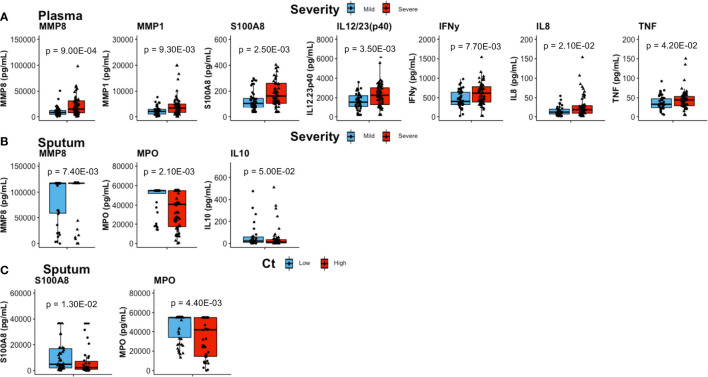
Comparisons of inflammatory mediator concentrations at baseline. **(A)** In plasma, patients with severe lung damage (n = 61) had higher levels of proinflammatory mediators than those with mild lung damage (n = 46). **(B)** Sputum levels of mmp8 were higher whilst MPO and IL10 were lower in patients with severe (n = 60) compared to mild lung (n = 36) damage. **(C)** Whereas sputum S100A8 was higher and MPO was lower in patients with high Mtb loads (n = 45) compared to low Mtb loads (n = 53). Boxes represent the first and third quartiles and horizontal bars within indicate median concentration. Whiskers indicate minimum and maximum values. Each dot represents one individual patient. P-values were obtained using the Wilcoxon signed rank test.

Plasma concentrations of MMP8, MMP1, S100A8, IL12/23(p40), IFNγ, IL8, and TNF were significantly elevated in patients with severe lung damage at baseline compared to those with mild damage (p = 9.00E−04, p = 9.30E−03, p = 2.50E−03, p = 3.50E−03, p = 7.70E−03, p = 2.10E−02, and p = 4.20E−02, respectively; **Figure 1A**).

Severe lung damage was associated with plasma MMP8, MMP1, S100A8, IL12/23(p40), and IFNγ (p = 3.00E−03, p = 1.40E−02, p = 1.80E−02, p = 1.00E−02, and p = 1.90E−02, respectively) even after adjusting for sex (**Supplementary Table 5** and **Supplementary Figure 2A**). With respect to Mtb burden at baseline, the only difference in plasma inflammatory mediator levels between high Mtb and low Mtb load groups was observed for S100A9 (p = 4.60E−02, adjusted for sex) (**Supplementary Table 5**).

In sputum, MMP8 (p = 7.40E−03) levels were higher in patients with severe lung damage at baseline (**Figure 1B**) however, this was not significant after adjusting for sex. TNF levels were associated with lung damage severity only after adjusting for sex (p = 5.70E−02 and p = 4.50E−02; unadjusted and adjusted for sex, respectively) (**Supplementary Table 6**). In contrast, baseline sputum levels of IL10 (p = 5.00E−02) and MPO (p = 1.20E−03) were significantly lower in severe lung damage compared to mild lung damage group (**Figure 1B**). For MPO, the association with lung damage was significant (p = 1.80E−02) even after adjusting for sex (**Supplementary Table 6** and **Supplementary Figure 2B**). Additionally, sputum MPO (p = 4.40E−03) and S100A8 (p = 1.30E−02) concentrations were significantly lower and higher, respectively in patients with high Mtb load compared to those with low Mtb load (**Figure 1C**). This association between MPO levels and Mtb load was significant (p = 2.10E−02) even after adjusting for sex (**Supplementary Table 6**). For whole blood stimulated samples (EC, PPD, WCL, and PMA), there was no significant difference in baseline inflammatory mediator levels between either severe and mild lung damage or high and low Mtb loads (not shown).

We also observed that sputum MMP1 (p = 2.70E−02) and plasma concentrations of MMP3, IL8, IL10, IL12/23(p40), GM-CSF, and TNF (p = 3.00E−03, p = 3.76E−02, p = 4.03E−02, p = 3.30E−02, p = 2.65E−02, and p = 3.36E−02, respectively) were higher in males compared to females (**Supplementary Table 1**). Interestingly, sputum MPO concentrations were higher females (p = 1.85E−02) (**Supplementary Table 1**).

### Changes in Neutrophil Mediator Concentrations Post-Treatment

The majority of pro-inflammatory mediators in plasma decreased during TB treatment except for MMP3, MPO, and IL8 (**Supplementary Table 2**). Compared to baseline, plasma levels were lower at month 2 and month 6, respectively for MMP1, MMP8, MMP9, S100A8, S100A9, TNF, IFNγ, GM-CSF, IL10, and IL12/23(p40) (**Supplementary Table 2**). In sputum, concentrations of MMP1, MMP3, MMP8, MMP9, and TNF were significantly lower at both month 2 and month 6, when compared with baseline (**Supplementary Table 2**).

Additionally, sputum GM-CSF (p = 5.50E−07), TNF (p = 2.10E−05), IFNγ (ns), S100A8 (ns), and MPO (ns) were higher at month 6 compared to baseline (**Supplementary Table 2**). Interestingly, the concentrations of these specific mediators in whole blood stimulated samples were also higher after treatment compared to baseline. Notably, this increase was significant at month 6 for GM-CSF [EC, p = 2.70E−02; PPD, p = 1.50E−09; WCL, p = 2.80E−05, and PMA, p = 6.70E−11], TNF [EC, p = 2.80E−02; PPD, p = 2.00E−03; WCL, p = 2.00E−04 and PMA, p = 2.00E−02], IFNγ [EC, (ns); PPD, p = 3.20E−08; WCL, p = 6.30E−03 and PMA, p = 1.00E−11], S100A8 [EC, (ns); PPD, (ns); WCL, p = 2.80E−02 and PMA, (ns)), MPO (EC, (ns); PPD, (ns); WCL, p = 9.10E−03 and PMA (ns)] and MMP9 [EC, (ns); PPD, p = 1.90E−08; WCL, p = 3.60E−02 and PMA, p = 5.40E−07] (**Supplementary Table 2**).

The decrease in plasma and sputum concentrations of these mediators at month 6 compared to baseline was more pronounced in patients with initially (at baseline) severe lung damage (**Supplementary Table 3**) or initially high Mtb loads (**Supplementary Table 4**). Interestingly, this decrease in concentrations was exclusive to the initially severe lung damage group for S100A8 (p = 4.61E−09), MMP9 (p = 1.26E−02), IL10 (p = 3.86E−04), TNF (p = 3.77E−06), IFNγ (p = 3.84E−07) and GM-CSF (p = 4.54E−05) levels in plasma; and for MMP1 (p = 1.90E−05), MMP8 (p = 4.99E−05) and TNF (p = 1.31E−03) levels in sputum (**Supplementary Table 3**). No such analogy was observed when groups defined by Mtb burden were considered (**Supplementary Table 4**).

**Figure 2:**
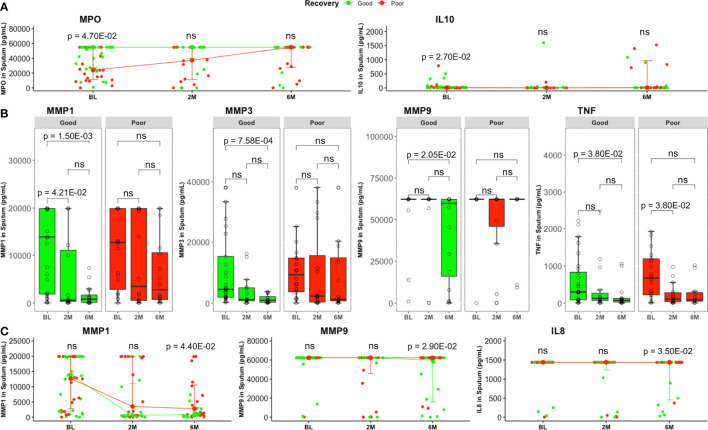
Comparison of sputum inflammatory mediator levels between good and poor lung recovery groups with treatment time. **(A)** MPO and IL10 were higher in good (BL, n = 29; 2M, n = 16; 6M, n = 16) compared to poor (BL, n = 22; 2M, n = 16; 6M, n = 16) lung recovery groups at baseline. Data represent median [IQR]. Differences between lung pathology groups at any given time point were compared using the Wilcoxon signed rank test. **(B)** Most MMP1, 3, 9, and TNF levels were significantly lower at month 6 compared to baseline in patients with good lung recovery but not in those with poor lung recovery. Boxes represent the first and third quartiles and horizontal bars within indicate median concentration. Whiskers indicate minimum and maximum values. Each dot represents one individual patient. Kruskal–Wallis test with Dunn’s post-test comparison was performed to analyse differences between time points. **(C)** At the end of standard TB treatment, MMP1, MMP9 and IL8 concentrations were still higher in patients with poor lung recovery compared to those with poor lung recovery. Groups were compared using the Wilcoxon signed rank test. Data represent median [IQR]. ns, not significant.

Patients with good lung recovery had higher baseline sputum MPO (p = 4.70E−02) and IL10 (p = 2.70E−02) levels compared to patients with poor recovery (**Figure 2A**). For MPO, the association with lung recovery was significant (p = 2.40E−02) after adjusting for sex (**Supplementary Table 6**). Additionally, logistic regression revealed significant associations between and lung recovery and levels of plasma MMP8 (**Supplementary Table 5**) and sputum TNF (**Supplementary Table 6**) after adjusting for sex (p = 3.90E−02 and p = 3.80E−02, respectively).

Additionally, sputum MMP1, MMP3, MMP9, and TNF levels decreased significantly (p = 1.50E−03, p = 7.58E−04, p = 2.06E−02, and p = 3.81E−02, respectively) from baseline to month 6 in patients with good lung recovery but not in those with poor recovery (**Figure 2B**). We also saw significantly higher levels of MMP1 (p = 4.40E−02), MMP9 (p = 2.90E−02) and IL8 (p = 3.50E−02) in sputum from patients with poor lung recovery compared to good lung recovery at month 6 (**Figure 2C**).

In whole blood stimulated supernatants, with the exception of IFNγ (WCL at BL; p = 1.60E−02), IL12/23(p40) (WCL at 6M; p = 4.80E−02) and S100A8 (EC at BL; p = 3.00E−02) concentrations which were higher in mild compared to severe lung damage; MMP1 (PPD at BL; p = 5.00E−03) which was higher in high compared to low Mtb load and; S100A8 (EC at BL; p = 3.00E−02) which was higher in low compared to high Mtb load, there were no other significant differences in inflammatory mediator levels of whole blood stimulated samples (EC, PPD and WCL) from patients in severe *vs* mild lung damage or high *vs* low Mtb loads at any time point (not shown).

**Figure 3:**
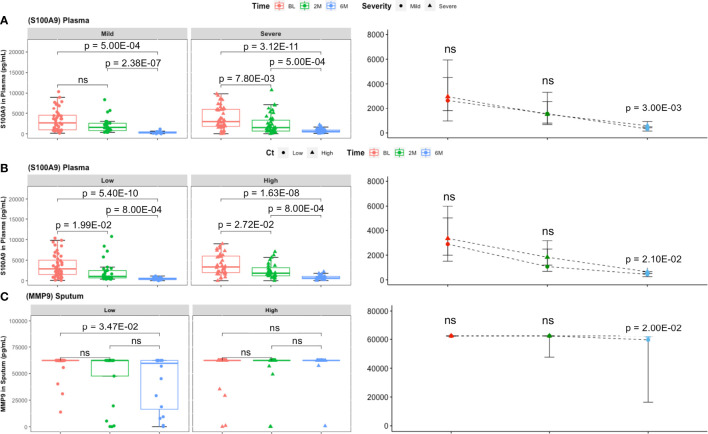
Comparison of inflammatory mediator levels between lung damage and Mtb burden defined groups with treatment time. **(A)** In plasma, the decrease in S100A9 concentrations was comparable between lung damage groups (mild: BL, n = 46; 2M, n = 25; 6M, n = 14 and severe: BL, n = 61; 2M, n = 42; 6M, n = 43) over time. However, at month 6, S100A9 was significantly higher in patients with initially severe lung damage compared to mild lung damage. **(B)** The same observation made between initially high Mtb and low Mtb loads with S100A9 levels being higher in the former at month 6. Boxes represent the first and third quartiles and horizontal bars within indicate median concentration. Whiskers indicate minimum and maximum values. Each dot represents one individual patient. Kruskal–Wallis test with Dunn’s post-test comparison was performed to analyse differences between time points. Differences between lung pathology groups at any given time point were compared using the Wilcoxon signed rank test. Data represent median [IQR]. **(C)** Sputum (low: BL, n = 46; 2M, n = 21; 6M, n = 16 and high: BL, n = 42; 2M, n = 22; 6M, n = 17) MMP9 levels were significantly lower at month 6 compared to baseline in patients with low but not in those with high Mtb loads. Additionally, S100A9 concentration at month 6 was significantly higher in patients with high Mtb compared to low Mtb load. ns, not significant.

Finally, S100A9 concentrations at month 6 were significantly higher in severe lung damage and high Mtb load groups compared to mild damage and low Mtb load groups, respectively (p = 3.00E−02, and p = 2.10E−02, respectively) at month 6 (**Figures 3A, B** **)**. Additionally, sputum levels of MMP9 declined significantly from baseline to month 6 in patients with low Mtb load (p = 3.20E−03) but not in patients with high Mtb load (**Figure 3C**). Moreover, these sputum concentrations of MMP9 remained significantly higher in the high Mtb load compared to the low Mtb load group at month 6 (p = 2.00E−02) (**Figure 3C**).

## Discussion

The aim of this study was to analyse neutrophil-associated soluble mediators in lung and blood samples from patients with different levels of lung pathology at baseline and recovery following treatment. We report stronger correlations between neutrophil counts and primarily neutrophil-derived mediators like MMP8, S100A8/A9, TNF, and GM-CSF as compared to other known TB-related inflammatory markers like IFNγ, IL10 or IL12/23(p40), for which neutrophils are not necessarily the major sources.

GM-CSF is a known neutrophil primer and MMP8 concentrations have previously been linked to clinical and radiological TB severity (44, 45), while S100A8/9 regulates CD11b expression and accumulation in chronic TB mouse models (28, 35). Our data supports these previous observations. We show that pre-treatment plasma levels of S100A8/9, MMP8 and GM-CSF correlate strongly with neutrophil counts and lung damage severity. Interestingly, while sputum levels of MMP8 correlate positively, MPO correlates negatively with lung damage and Mtb burden at baseline. This suggest that lung pathology results from increased systemic and pulmonary inflammation. It also hints that MPO could dampen the inflammatory response in ATB, thereby preventing excessive bacterial load and lung damage. We recently revealed that neutrophil subsets are associated with protective or detrimental effects on the severity of TB-linked lung pathology (13). Gideon and collaborators also showed pro- (IFNγ) and anti-inflammatory (IL10) cytokine expression by different neutrophil subsets in granulomas from Mtb-infected cynomolgus macaques (24), suggesting an immunoregulatory function of neutrophils in TB granulomas. Also, neutrophil elastase dissociation is triggered by reactive oxygen species (ROS) in an MPO-dependent manner during NETosis (46, 47), suggesting that NETs are involved in an MPO-related protective mechanism. Additionally, Mtb control by HIV-coinfected macrophages is enhanced by apoptotic neutrophils in *via* an MPO-dependent process (48). Whilst MPO inhibition is reported to block Mtb-induced necrotic cell death (49), MPO-deficient mice develop severe lung inflammation following exposure to zymosan (37). In fact, a recent review details the numerous protective and harmful functions of MPO in human disease (50). Whilst IL10 is released by several immune cell types during TB and monocytes/macrophages also produce MPO, neutrophil granules are the main source of MPO (48). This to our knowledge, is the first report of an MPO-related beneficial role in TB-related lung pathology and recovery.

The current literature overwhelmingly supports a detrimental effect of neutrophils on lung pathology in TB patients, however, some neutrophil subsets are protective. Specific neutrophil subsets were associated with protective outcomes against TB lung pathology suggesting that the variations observed in disease outcomes may be driven by different immunomodulatory mediators or interactions with other immunocytes (13). Additionally, a neutrophil-driven regulatory effect is not unheard of. In fact, neutrophils (*via* CD11b-dependent responses) and endothelial cells have been shown to cooperate in detection and capture of pathogens in lung capillaries (51). Also, neutrophils play a central role in controlling human metapneumovirus-induced inflammation by regulating γδ T cell recruitment to the lung (52). Meanwhile, neutrophils were found to suppress of lymphocyte function by secreting MPO and hydrogen peroxide (53) and Mtb-specific stimulation of neutrophils inhibits antigen-specific T-cell production of IFNγ (24). More recently, this neutrophil-related immunosuppressive function on lymphocytes has been attributed to hyper-segmented subsets (7) and to the neutrophil-like MDSCs (54, 55) (in cancer (56, 57), leukaemia (58) and lately TB (9, 59). While the immunosuppressive roles of MDSCs on Mtb pathogenesis are still under investigation, recent experimental models show benefits in limiting their accumulation during TB HDTs (25, 60). We suggest that MPO could be protective against TB progression and lung damage.

We understand that functional analysis of neutrophils is technically difficult considering that they are short-lived, easily activated by laboratory processing methods and cannot be cryopreserved. Nevertheless, we support future investigation of mechanistical pathways that promote the secretion of these mediators or increased production of neutrophil subsets that produce them to achieve desirable outcomes during ATB. We could not confirm the link between MPO and bacterial burden using Mtb killing assays *in-vitro*. However, prospective studies within the TB sequel project are being designed to achieve that by assessing the levels of these mediators in the presence/absence of TB-targeted HDTs. We also recommend studies using isolated neutrophils from patients within these different lung pathology and gender groups to address this gap in knowledge (potentially also in animal models).

Variability in immune responses between genders have been linked to: specific immune cell types, age, levels of sex hormones, environmental factors (e.g., nutritional status or microbiome composition) and disease states (61). In accordance with previous studies (62, 63), ATB prevalence in our Gambian cohort is higher in males. Also similar to previous studies on chronic inflammatory diseases (64, 65), we observe that the pro-inflammatory response in males is higher than that in females. We report higher plasma levels of notoriously pro-inflammatory mediators like TNF, MMP3, GM-CSF, and IL8 in males compared to females. This is in accordance with the observation that male neutrophils are more responsive to LPS and IFNγ stimulation than female neutrophils; with the former expressing higher levels of toll-like receptor 4 (TLR4) and producing more TNF (66). Meanwhile, we also observe higher sputum MPO levels in females, supporting the idea that increased MPO concentration is linked to suppressed inflammation.

Moreover, patients showing good lung recovery had higher sputum MPO concentrations at baseline. In contrast, MMP8 and sputum TNF levels were positively associated with poor recovery after adjusting for sex. These suggest that MPO, MMP8, and TNF play a considerable role in determining the degree of recovery from severe TB-related lung damage after treatment. It also supports future investigation of these mediators as proxies for predicting lung recovery following injury.

As treatment progresses, sputum and plasma concentrations of MMP1, 8, 9 and plasma levels of S100A8/9 and MMP3 decrease rapidly, suggesting that the neutrophil-related inflammatory response and matrix-degrading activity are not only fuelled by MMPs ((32, 33, 67)) and calprotectin (28) activity but also potentially resolved by variations in levels of these mediators with treatment. In contrast, sputum levels of IL8 and MPO remain fairly constant, suggesting that variations in neutrophil (and potentially monocyte) recruitment and overall activity during ATB treatment may be more complexly regulated. This is supported by our other observation that the decrease in concentrations of S100A8, MMP9, IL10, TNF, IFNγ, and GM-CSF in plasma and MMP1, MMP8, and TNF in sputum are exclusive to the severe lung damage group; suggesting that these inflammatory mediators are major contributors to severe TB-related lung pathology pre-treatment. The fact that no such analogy was observed when groups defined by Mtb burden were considered also supports the idea that high Mtb loads are not necessarily ascribed to severe lung damage outcomes (42).

Post-therapy, we observed high plasma S100A8 levels in severe compared to mild lung damage group, meanwhile plasma S100A8 and sputum MMP9 were significantly higher in patients with initially high Mtb load compared to the initially low Mtb load group. S100A9 and MMP9 are neutrophil-derived mediators, suggesting that severe lung damage at presentation may contribute to heightened residual neutrophil activity after treatment. Also, post-treatment levels of sputum MMP1, MMP9, and IL8 were higher in patients with poor lung recovery compared to those with good lung recovery. This suggests that unresolved lung damage is linked to continuous neutrophil activity and persistent leucocyte infiltration in the lungs post-treatment. While, previous studies suggested that for patients with severe lung damage, recovery may only begin many months after the end of standard ATT (68, 69), a possible reason for this was not provided. This, to our knowledge is the first report of several major neutrophil-derived mediators (in plasma and sputum) being directly linked to TB lung pathology and unresolved lung damage. Furthermore, higher levels of MMP1, MMP9, and IL8 in sputum from patients with poor compared to good lung recovery at month 6 suggest that poor lung recovery results from continuous neutrophil activity and persistent leucocyte infiltration in the lungs even after treatment completion.

For whole blood stimulated supernatants (notably with H37Rv whole cell lysate), the increased levels of GM-CSF (also increased in sputum), TNF (also increased in sputum), IFNγ, S100A8, MPO and MMP9 after treatment compared to baseline hint at an enhanced sensitivity of immune cells to pathogen stimulation. Previous studies have reported lower cytokine production by T-cells pre-treatment, suggesting that continual pathogen stimulation results in T-cell exhaustion which is then restored after treatment (reviewed in (70). To our knowledge, this is the first report of increased concentrations of major neutrophil-derived mediator levels in ATB post-treatment compared with pre-treatment levels. These suggest that chronic TB could directly (or indirectly, *via* T-cell exhaustion which leads to either higher levels of immune-inhibitory molecules like PD-1 (71, 72) and TIM3 (73) or reduced release of innate immune cell activators like IFNγ and TNF) result in reduced neutrophil activity pre-treatment. It also highlights the need to monitor the impact of neutrophil interactions with other immunocytes on TB pathogenesis. Finally, we suggest that toll-like receptor (TLR)-mediated pathogen sensing by lung epithelial/innate immune cells, MPO-regulated NET formation, neutrophil migration/activation following increased secretion of inflammatory mediators (e.g., S100A8/9, MMP8, GM-CSF, TNF, IFNγ and potentially IL17/IL17R, RANTES, IL6, ICAM1, etc.) and ROS release/NADPH-dependent leucocyte recruitment (74–76) are immune pathways potentially involved in balancing the neutrophilic inflammatory response during ATB.

## Conclusion

We show that S100A8/9 and MMP8 contribute to increased lung damage and that MPO acts as an anti-inflammatory agent which potentially regulates TB-related lung pathology and promotes lung recovery. We also suggest that increased MPO-mediated immunosuppression could limit lung pathology in females. Treatment results in decreased inflammation characterised by lower sputum and plasma concentrations of neutrophil-derived pro-inflammatory mediators especially in patients with severe lung pathology (but not High Mtb load) at presentation. We hereby highlight the relationship between neutrophil-derived inflammatory mediator levels and radiological disease severity irrespective of Mtb burden. We also report that S100A8/9 and other neutrophilic mediators like MMP9 and IL8 may be responsible for unresolving lung damage in patients with poor lung recovery. Finally, we recommend targeting S100A8/9, MMP8, and MPO for developing host-directed therapies against TB-induced lung pathology and to promote recovery.

## Data Availability Statement

The raw data supporting the conclusions of this article will be made available by the authors, without undue reservation.

## Ethics Statement

The studies involving human participants were reviewed and approved by the Medical Research Council/Gambia government joint ethics committee (SCC1523). The patients/participants provided their written informed consent to participate in this study.

## Author Contributions

CN: Conceptualisation, Data curation, Formal analysis, Investigation, Methodology and writing of manuscript. OO: Patient recruitment and follow-up, clinical data, and review of manuscript. SD: Data Management. JM: Assisted with wet-lab experiments. IS: Assisted with wet-lab experiments. SC: Funding acquisition and review of manuscript. AB: Data curation and Formal analysis. AR: Conceptualisation, Funding acquisition, data analysis, and review of manuscript. CG: Supervision, Methodology, and manuscript review. JS: Supervision, Conceptualisation, Data curation, Methodology, Funding acquisition, and review of manuscript. All authors contributed to the article and approved the submitted version.

## Funding

This work was supported by a PhD student stipend from TB Sequel (grant number 66.3010.7-002.00) funded by the German Ministry for Education and Research (BMBF).

## Conflict of Interest

The authors declare that the research was conducted in the absence of any commercial or financial relationships that could be construed as a potential conflict of interest.

## Publisher’s Note

All claims expressed in this article are solely those of the authors and do not necessarily represent those of their affiliated organizations, or those of the publisher, the editors and the reviewers. Any product that may be evaluated in this article, or claim that may be made by its manufacturer, is not guaranteed or endorsed by the publisher.
