# Supplementary material for: Major Neutrophil-Derived Soluble Mediators Associate With Baseline Lung Pathology and Post-Treatment Recovery in Tuberculosis Patients
Source: Front Immunol. 2021 Nov 23;12:740933. doi: 10.3389/fimmu.2021.740933 (PMC8650718; doi:10.3389/fimmu.2021.740933)
Supplement: Supplementary file 1 [file DataSheet_1.pdf]

Supplementary Figures

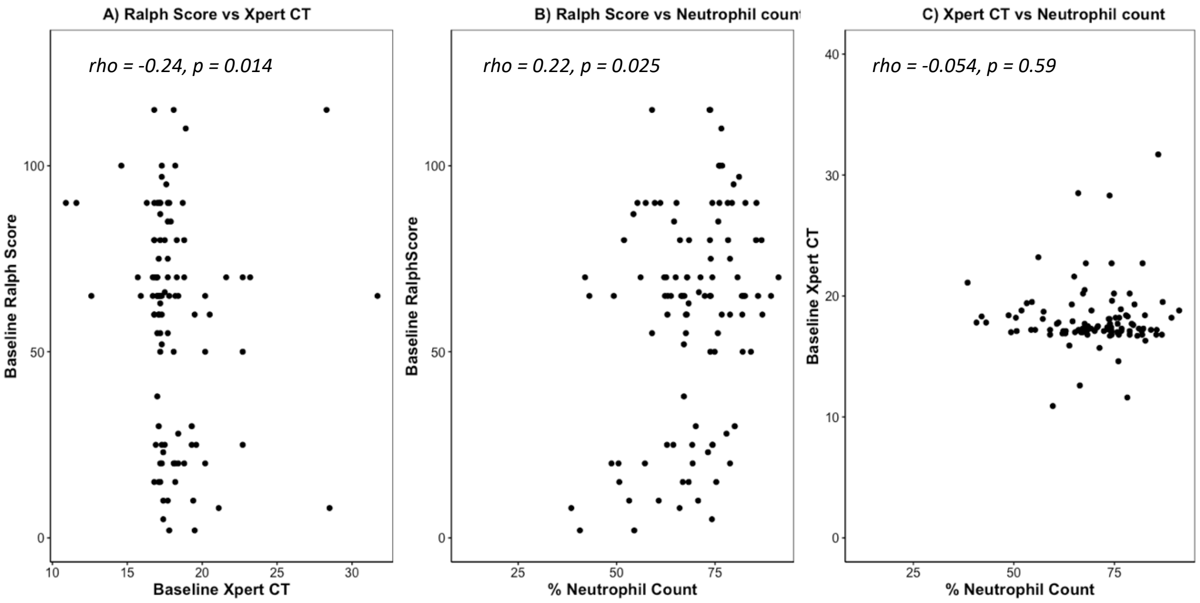

Supplementary Fig. 1: Spearman correlation curves between. A) baseline Ralph score and Xpert Ct values B) baseline Ralph score and neutrophil counts and C) baseline Xpert Ct values and neutrophil counts. Correlations were performed using Spearman rank-order correlation coefficient.

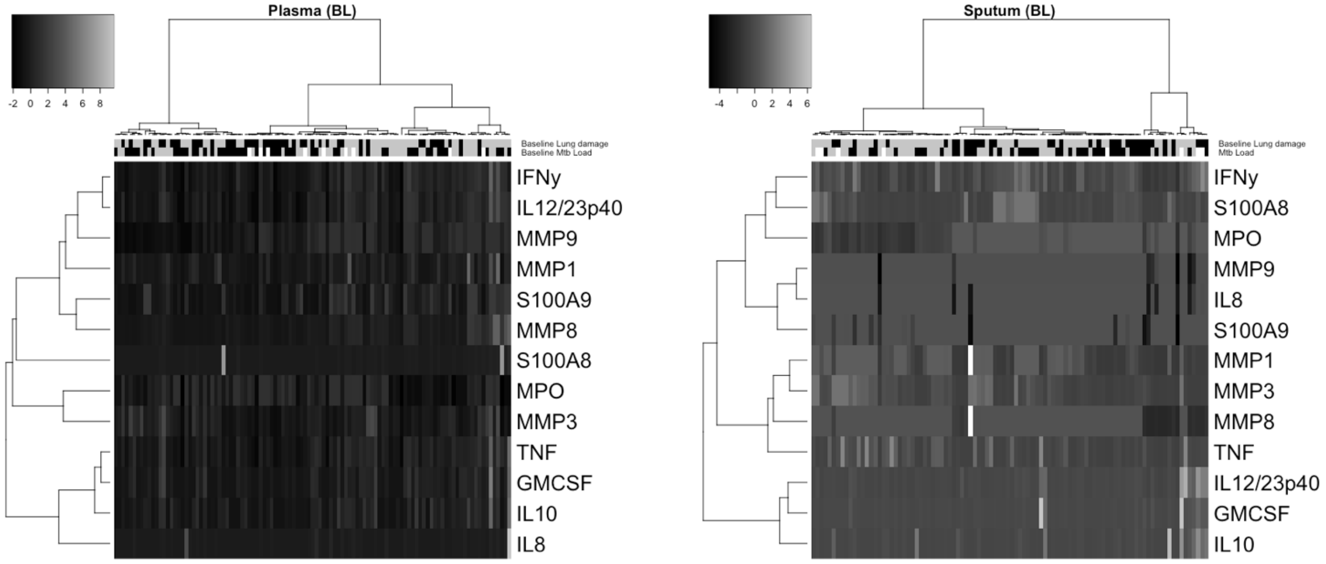

Supplementary Fig. 2: Inflammatory mediator level heatmap representing baseline inflammatory mediator concentrations for ATB patients. Showing severe lung damage and high Mtb load (grey) or mild lung damage and low Mtb load (black), respectively in: A) plasma and B) sputum. White represents patients that could not be classified into either good or poor recovery groups due to missing month 6 CXR scores (NA). A non-supervised two-way hierarchical cluster analysis (Ward's minimum variance method) was employed to grade TB disease severity, using the Ralph score and Xpert Ct values.
